# Identifying critical higher-order interactions in complex networks

**DOI:** 10.1038/s41598-021-00017-y

**Published:** 2021-10-28

**Authors:** Mehmet Emin Aktas, Thu Nguyen, Sidra Jawaid, Rakin Riza, Esra Akbas

**Affiliations:** 1 Department of Mathematics and Statistics, University of Central Oklahoma, Edmond, OK 73034 USA; 2 Department of Computer Science, University of Central Oklahoma, Edmond, OK 73034 USA; 3 Department of Computer Science, Oklahoma State University, Stillwater, OK 74074 USA

**Keywords:** Mathematics and computing, Applied mathematics, Computational science, Computer science, Information technology, Software, Statistics

## Abstract

Diffusion on networks is an important concept in network science observed in many situations such as information spreading and rumor controlling in social networks, disease contagion between individuals, and cascading failures in power grids. The critical interactions in networks play critical roles in diffusion and primarily affect network structure and functions. While interactions can occur between two nodes as pairwise interactions, i.e., edges, they can also occur between three or more nodes, which are described as higher-order interactions. This report presents a novel method to identify critical higher-order interactions in complex networks. We propose two new Laplacians to generalize standard graph centrality measures for higher-order interactions. We then compare the performances of the generalized centrality measures using the size of giant component and the Susceptible-Infected-Recovered (SIR) simulation model to show the effectiveness of using higher-order interactions. We further compare them with the first-order interactions (i.e., edges). Experimental results suggest that higher-order interactions play more critical roles than edges based on both the size of giant component and SIR, and the proposed methods are promising in identifying critical higher-order interactions.

## Introduction

Diffusion on networks is an important concept in network science observed in many situations such as information spreading and rumor controlling in social networks, disease contagion between individuals, and cascading failures in power grids. Depending on various factors, some elements in a network play critical roles in the diffusion process as they affect the network structure and functions significantly more than others. For example, in social networks, one can spread messages in the network quickly through the critical nodes^1^; in epidemic networks, one may reduce the diffusion of epidemic by controlling influential nodes^2^. Therefore, identifying critical (influential) nodes and edges has practical importance in network science. There are many studies in the literature for the critical node detection problem in networks. While some studies are based on degree of nodes such as degree centrality^3^ and H-index^4^, some use paths in networks such as closeness centrality^5^ and betweenness centrality^6^. Others use eigenvectors of graphs such as PageRank^7^ and DFF centrality^8^. In addition, some researchers use node deletion or contraction to distinguish the importance of nodes^9,10^.

Moreover, edges in networks also play a critical role in information diffusion^11–19^. For example, the identification of critical edges can be helpful to analyze the vulnerability in electrical transmission networks. Many researchers focus on finding critical edges based on network topology. For instance, the authors in^11^ use the degree values of two nodes connected by an edge to measure the importance of that edge. In^12–14^, the authors use the betweenness centrality of edges to detect critical edges. In other words, they assume that edges connecting two connected components are important. There are also other studies that use flow/reachability^15,16^, bridgeness^19^, neighbors^17^, and clique degrees^18^ to measure the edge importance.

On the other hand, as we see in different real-world applications such as human communication, chemical reactions, and ecological systems, interactions can occur between not only two nodes as pairwise interactions, i.e., edges, but also between three or more nodes^20^, which are described as *higher-order* interactions. Hypergraphs are used to model higher-order interactions in complex systems where entities are represented as nodes, and higher-order interactions among them are represented as hyperedges. For example, in coauthorship networks, nodes represent authors and hyperedges represent articles. In drug–drug interaction networks, nodes represent substances that make up the drug, and hyperedges represent drugs. Moreover, hyperedges play a critical role in information diffusion. For instance, in order to find the most influential article in a coauthorship network, we need to find the most influential hyperedge. Similarly, in a social network, manufacturers intend to detect influential hyperedges for promoting their products to maximize the number of influenced customers.

There are a few studies that explore other critical structures in graphs, such as critical groups of nodes and edges. But, these studies do not consider higher-order interactions in hypergraphs. In^21^, the authors study the problem of finding the most and least influential cliques of fixed size in graphs based on group degree, group closeness, and group betweenness centralities. However, the proposed methods only detect the most critical group of a fixed size and are unable to compare groups of any size. In^22^, the authors use the group betweenness centrality to find the most influential group of nodes and a general group of graph elements (containing nodes and edges) of a fixed size. However, they are not able to compare higher-order interactions but rather groups (not necessarily connected). In^23^, the same authors find the most influential group of nodes using the group closeness centrality and further, identify a subset of critical edges whose removal maximally degrades the closeness centrality of those vertices. But again, this paper is not focusing on higher-order interactions. In^24,25^, the authors find influential higher-order interactions based on closeness and *H*-closeness centrality only. Furthermore, the authors in^26^ study the problem of detecting initially-influenced seed users of a fixed size in a directed hypergraph. Similarly, the authors in^27^ detect the smallest set of initially influenced nodes in hypergraphs such that all the users are influenced at the end of the influence diffusion process. But these studies only focus on nodes rather than higher-order interactions.

On the other hand, modeling diffusion in hypergraphs via Laplacians, which is the key concept to compute the centralities, is not a simple task. This is because hyperedges can include more than two vertices, and edge incidence and vertex adjacency are set-valued in hypergraphs. To handle this issue, researchers limit their attention to uniform hypergraphs, where hyperedges have the same cardinality^28,29^. But this is not realistic since real-world hypergraphs are almost never uniform. As another approach to this issue, researchers reduce non-uniform hypergraphs to graphs using the line graph and clique expansion. However, these reductions unsurprisingly result in information loss and are unable to uncover hypergraph structure^30,31^. Some studies study random walks on hypergraphs for modeling diffusion, but much of them only consider uniform hypergraphs^32–34^. In the non-uniform case, these random walks are equivalent to a random walk on the graph clique expansion of the hypergraph^35,36^.

As another solution, Horak et al.^37^ defines the simplicial Laplacians for the hypergraphs with the simplicial complex structure, i.e., subsets of hyperedges are also hyperedges. However, these Laplacians have three critical issues. First, it is defined only for hypergraphs with the simplicial complex structure, i.e., subsets of hyperedges are also hyperedges, which is often not the case in real-world hypergraphs. Second, for a hyperedge of size *k*, the simplicial Laplacian models the diffusion only *through* the hyperedges of sizes $k-1$ and/or $k+1$. However, in the diffusion framework, information on a hyperedge can diffuse through other hyperedges regardless of their sizes. Third, when we use the simplicial Laplacians in modeling diffusion, we need to assume that information only diffuses *between* fixed size hyperedges. However, a hyperedge can affect other hyperedges regardless of their sizes. Hence, there is a need for more broad and general hypergraph Laplacians to model diffusion to detect the critical higher-order interactions.

In this report, to address these limitations, we propose two new hypergraph Laplacians based on the diffusion framework that allow us to find the influential higher-order interactions in a hypergraph of any size and with any desired classical centrality measure; one is based on diffusion between *fixed size* hyperedges, and the other is based on diffusion between *all* hyperedges. The previously developed hypergraph Laplacians are only defined for special hypergraphs, and more importantly, neglect the relations between hyperedges. Thanks to the proposed Laplacians, we can model the complete relations between hyperedges of any size. Next, using the relations between hyperedges, we extend four graph centrality measures, namely DFF ($H_{DFF}$), degree ($H_{Deg}$), betweenness ($H_{Btw}$), and closeness ($H_{Cls}$), to hypergraphs and rank higher-order interactions based on these measures. One can also similarly extend other classical centrality measures, but we believe working with four centralities would be enough to show the effectiveness of the proposed method. For evaluation, we experiment on several undirected real-world network datasets and evaluate the performance using the size of giant component^38^ and Susceptible-Infected-Recovered (SIR) simulation model^39^. We further compare influential higher-order interactions with the first-order interactions (i.e., edges) to show the effectiveness of using higher-order interactions. The experimental results suggest that higher-order interactions are more influential than edges based on both the size of giant component and SIR, and our methods are quite promising in finding influential higher-order interactions.

## Results

In this section, we first describe the datasets we use in our experiments. Then, we explain the size of giant component measure and how we model SIR on hypergraphs using the proposed Laplacians for evaluation. Next, we generalize four classical graph centrality measures, namely DFF, degree, betweenness, and closeness, to hypergraphs and present results on the datasets.

### Data description

In our experiments, we use four undirected real-world networks to evaluate the effectiveness of redefined centrality measures using hypergraph Laplacians (see Table 1 for the networks’ statistics). (1) Enron: each vertex represents the email address of a staff member at Enron. A hyperedge represents all the recipients, including the sender, of an email sent between the Enron staff. (2) High school: this dataset is made from a network of high school students in Marseilles, France. A vertex is a student, and a hyperedge is a set of students in close contact with each other. (3) Primary school: this dataset is made from a network of primary school students and teachers. A vertex is a student or a teacher, and a hyperedge is a set of students and/or teachers in close contact with each other. (4) NDC-classes: a vertex is a pharmaceutical class label used to classify a certain property of a drug. The network of drugs is taken from the National Drug Code Directory. A hyperedge is drug with many class labels. These datasets can be found in^40^.

**Table 1:** Basic properties of the real-world datasets we use are provided here.

Dataset	|*V*|	|*H*|	|*E*|	$\langle k\rangle$	$k_{max}$
Enron	143	1630	1800	106.3	19
High school	327	8264	5818	81.9	6
Primary school	242	13041	8317	197.2	6
NDC-classes	1149	2330	6222	62.5	25

|*V*| is the number of vertices, |*H*| is the number of hyperedges, |*E*| is the number of edges in the projected graph, $$\langle k\rangle$$ is the average weighted degree, and $$k_{max}$$ is the maximum hyperedge size.

### Evaluation metrics

For the evaluation of the proposed methods, we use the size of giant component and the SIR simulation model. In both methods, we compare higher-order interactions with the first-order interactions (i.e., edges) to show how higher-order interactions improve the experimental results. To compare with the first-order interactions, we project hypergraphs into graphs by representing pairwise relations in hyperedges with edges. We further investigate the effect of parameter setting in the SIR models for each centrality measure of higher-order interactions here.

#### The size of giant component $\sigma$

In this evaluation method, we first rank the interactions from the most influential to the least for each centrality. We then remove the interactions from the network one by one starting from the most to the least, and calculate the size of the giant (largest) connected component. A more effective method should have a faster fall in its size of giant curve and the area under its curve should be smaller.

**Figure 1:**
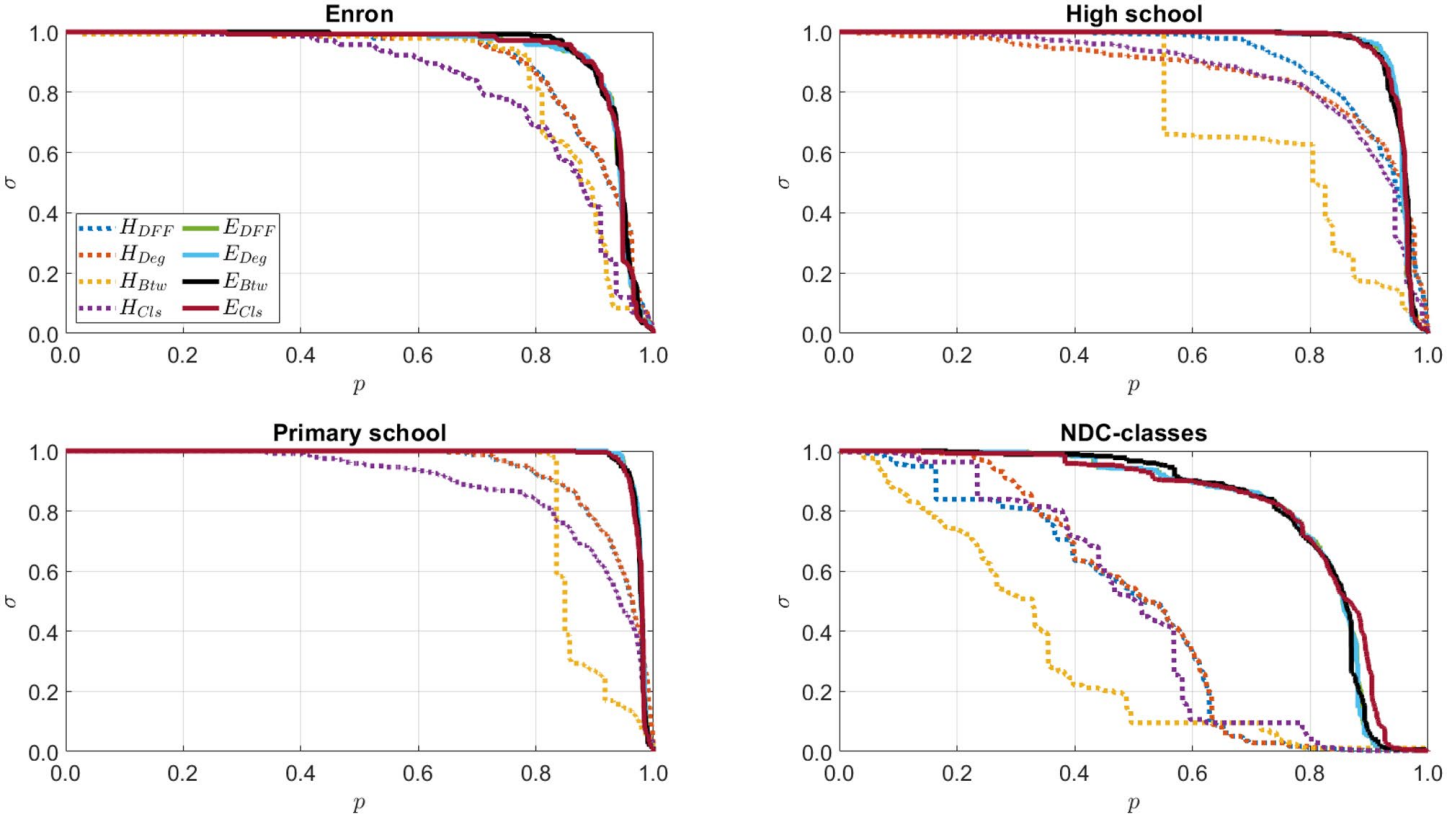
The size of giant component, $\sigma$, over varying ratio, *p*.

The results are shown in Fig. 1 and Table 2. As we see in Fig. 1, the influential higher-order interactions have all faster falls than the influential edges. This means the influential higher-order interactions break down the networks more quickly. The reason is whenever we remove an edge between two vertices, these vertices are likely still connected through other edges. On the other hand, when we remove a higher-order interaction, it is less likely to have connections between its vertices. More specifically, $H_{Cls}$ and $H_{Btw}$ curves in Enron and Primary school, and High school and NDC-classes networks fall the fastest, respectively.

For a more detailed comparison, we also calculate the area under the size of the giant component curves in Fig. 1. The smaller area means the better performance. As we see in Table 2, the higher-order interactions all have smaller areas than edges. Furthermore, in general, $H_{Btw}$ outperforms other hypergraph centralities.

**Table 2:** Area under the size of giant component curves in Fig. 1 for edges (*E*) and higher-order interactions (*H*).

	Enron		High school		Primary school		NDC-classes	
	*E*	*H*	*E*	*H*	*E*	*H*	*E*	*H*
$\mathbf {DFF}$	0.928	**0.892**	0.953	**0.908**	0.977	**0.935**	0.804	**0.469**
$\mathbf {Degree}$	0.928	**0.893**	0.953	**0.867**	0.977	**0.936**	0.803	**0.495**
$\mathbf {Betweenness}$	0.934	**0.859**	0.953	**0.755**	0.976	**0.873**	0.807	**0.321**
$\mathbf {Closeness}$	0.930	**0.826**	0.953	**0.864**	0.975	**0.886**	0.810	**0.483**

The smaller values means the better performance. The cells for the best (smallest) value in each row for each dataset is typed bold.

#### SIR model

We use the Susceptible-Infected-Recovered (SIR) simulation model on networks as an evaluation metric to objectively analyze the effect of higher-order interactions in diffusion between nodes. In the SIR model, each node is classified as a Susceptible (S), Infected (I), or Recovered (R) at any given moment. A selected node is initially infected, and the rest of the network is susceptible to be infected. In each propagation, the infected node can infect its neighboring nodes with probability $\mu$. As this process is repeated, infected nodes can recover with probability $\beta$ and are not susceptible to be infected again. The process stops when there is no infected node present in the network or after 500 propagations if there is still. The diffusion level is measured by the total number of nodes that were infected, including nodes that recovered, after all propagations are complete. A greater number means a greater spreading ability and a greater influence on diffusion.

In our experiments with the SIR model, we set the infection rate $\mu$ based on $\mu _c$, where $\mu _c=\frac{\langle k\rangle }{\langle k^2\rangle -\langle k\rangle }$, as derived from^41^, and $\langle k\rangle$ is equal to the average weighted degree of the network. We set the infection rate $\mu$ as $\mu _c$ multiplied by a factor depending on the different results we obtain, as explained in the following paragraphs. Furthermore, for the infection rate, we can also consider the weights of interactions. As we explain in the methods section, the proposed Laplacian between vertices assigns weight to each interaction as the number of shared hyperedges between two vertices. In this paper, we set the infection rate of an interaction of weight $w > 0$ to $\mu _w=1-(1-\mu )^w$, following^42^. For simplicity, the recovery rate is set as $\beta =1$. The experiment is run 100 times for each dataset, and the average of the 100 trials is taken to obtain more reliable results.

As^18^ suggests, we use the normalized final effected scale for evaluation, which is defined as

$$\begin{aligned} \displaystyle F(u)=\frac{n_u}{n} \end{aligned}$$

where $n_u$ is the number of affected nodes when node *u* is infected, and *n* is the total number of nodes. To compute the influence of higher-order interactions, we calculate the average influence of all nodes after removing a certain fraction of hyperedges as the following diffusion index

1 $$\begin{aligned} \displaystyle R_s=\frac{F_1 - F_2}{F_1} \end{aligned}$$

where $F_i$ is the average final infected scale of all nodes, i.e., $F_i=\frac{1}{n}\sum _{u \in V} F(u)$ for $i \in \{1,2\}$, and $F_1$ and $F_2$ are results of the original network and the network after removing *p* of higher-order interactions respectively. The larger diffusion index means the removed interactions are more influential.

In our experiment, we first rank the first-order and higher-order interactions from the most influential to the least for each centrality and divide them into 50 equal parts, i.e., the first part includes the top 2% influential interactions and the last part includes the bottom 2% influential interactions. In each iteration, we only remove one part among the 50 parts (other 49 parts are remaining) and calculate the diffusion index $R_s$ (1). We repeat this process for each part. Since the larger diffusion index means the removed interactions are more influential, we expect to see that the first part that corresponds to the top 2% influential interactions has the largest diffusion index, and the diffusion index decreases monotonically till the last part. To measure this correspondence, we use the spearman correlation coefficients. We first sort these diffusion indices from the biggest to the smallest. Now, we have two sequences for each centrality; one is coming from centrality scores and the other is coming from diffusion indices. To check the effectiveness of each centrality, we take the sequence coming from the diffusion indices as the ground truth and find the spearman correlation coefficients between this sequence and the sequence coming from centrality scores.

As we see in Table 3, the proposed hypergraph centrality measures can find the influential higher-order interactions in diffusion effectively. All the correlations are high for the Enron dataset and are about 90%, except for 85% for $H_{DFF}$. In the High school dataset, all of the centrality measures’ rankings are highly correlated with the SIR findings: they are all about $98 \%$. It is the same for the Primary school dataset, except $H_{Btw}$. The correlations are a little lower for the NDC-classes dataset than the other datasets (it is in between 76–80% for all but about 55% for $H_{Btw}$). The reason is that since the ratio between the number of nodes and hyperedges is low in this dataset, the infection rate becomes relatively small. This makes diffusion difficult in the SIR simulations, and as a result, it makes it slightly more difficult to see the effects of removing higher-order interactions. In general, $H_{DFF}$, $H_{Deg}$, and $H_{Cls}$ provide similar effectiveness, and $H_{Btw}$ is slightly less effective on the NDC-classes and Primary school datasets. We should also note here that our main goal here is not to compare these proposed hypergraph centralities but to show how effective they are in finding influential higher-order interactions. As we see in Table 3, overall, they are quite effective in finding influential higher-order interactions.

Furthermore, as we see in Table 3, the first-order interactions have negative or very small spearman correlation coefficients, which shows they as critical as higher-order interactions in the diffusion process on networks. This is due to the same reason as we explain in the size of giant component.

**Table 3:** Spearman correlation coefficients between the ranking scores and the diffusion indices for edges (E) and higher-order interactions (H).

	Enron		High school		Primary school		NDC-classes	
	*E*	*H*	*E*	*H*	*E*	*H*	*E*	*H*
$\mathbf {DFF}$	− 0.3720	**0.8587**	− 0.1032	**0.9893**	0.0190	**0.9764**	− 0.2099	**0.7943**
$\mathbf {Degree}$	− 0.4022	**0.9025**	− 0.3499	**0.9779**	− 0.1821	**0.9805**	− 0.1708	**0.7684**
$\mathbf {Betweenness}$	− 0.4448	**0.9067**	− 0.1360	**0.9839**	− 0.2830	**0.8364**	0.2948	**0.5432**
$\mathbf {Closeness}$	− 0.2964	**0.9055**	0.0145	**0.9854**	− 0.3438	**0.9558**	0.0099	**0.7811**

The results are averaged over 100 independent implementations with $\mu /\mu _c=1.5$. The cells for the best value in each row for each dataset is typed bold.

We further investigate the effect of parameter setting in the SIR models for each centrality measure of higher-order interactions with two experiments. First, we fix the infection rate and vary the ratio of the removed influential higher-order interactions, Second, we fix the ratio of the removed higher-order interactions and vary the infection rate.

**Figure 2:**
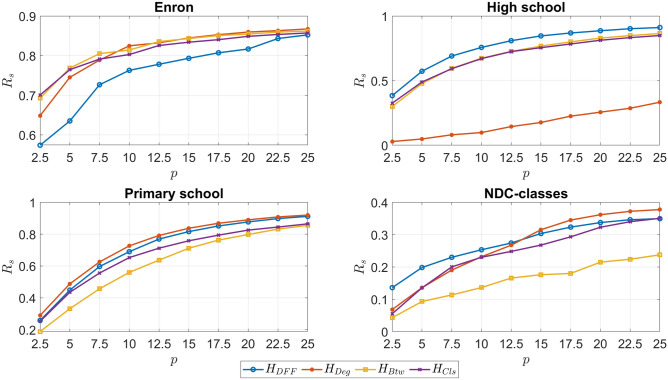
Varying ratio of hyperedges through the implementation of the SIR Model. Here the infection rate is kept constant while the influential hyperedges are obtained by using each of the centrality measures. A higher diffusion index ($R_s$) determines the effectiveness of each of these methods.

As the first experiment, we fix the infection rate at $\mu =1.5\mu _c$ and vary the ratio of the removed influential higher-order interactions in Fig. 2. The graphs are plotted with the diffusion index $R_s$ on the *y*-axes and the varying ratio of hyperedges (*p*) on the *x*-axes. When analyzing the graph for the Enron dataset, it can be inferred that degree, betweenness, and closeness are seen to overlap each other and possesses a higher diffusion index $R_s$ in comparison to DFF centrality. Therefore, it can be concluded that for the Enron dataset, degree, betweenness, and closeness centralities can be considered to be almost equally effective due to a consistently high $R_s$ value with the increasing ratio of hyperedges. For the High school dataset, it can be observed that DFF centrality is the most effective of the four centrality measures with betweeness and closeness centralities overlapping and following the same set of values for $R_s$ with an increase in the varying ratios. In contrast, the degree centrality proved to be least effective for its low $R_s$ value below 0.5. Primary school dataset has the most consistent increase in the value of diffusion index $R_s$ for all the centrality measures as there are little or no anomalies within the curves plotted for the four different centrality measures. The degree and the DFF centralities are considered the most effective centralities as they both yield the highest value of $R_s$ in comparison to betweenness and closeness centralities. Finally, for the NDC-classes dataset, it can be seen that initially the DFF centrality had a higher diffusion index ($R_s$) in comparison to the degree centrality. Still, after the ratio (*p*) increases above 12.5, the degree centrality proves to be more effective than the DFF centrality and the other two centrality measures. In conclusion, all the four centrality measures proved to be effective with varying ratios of higher-order interactions used within this SIR model.

**Figure 3:**
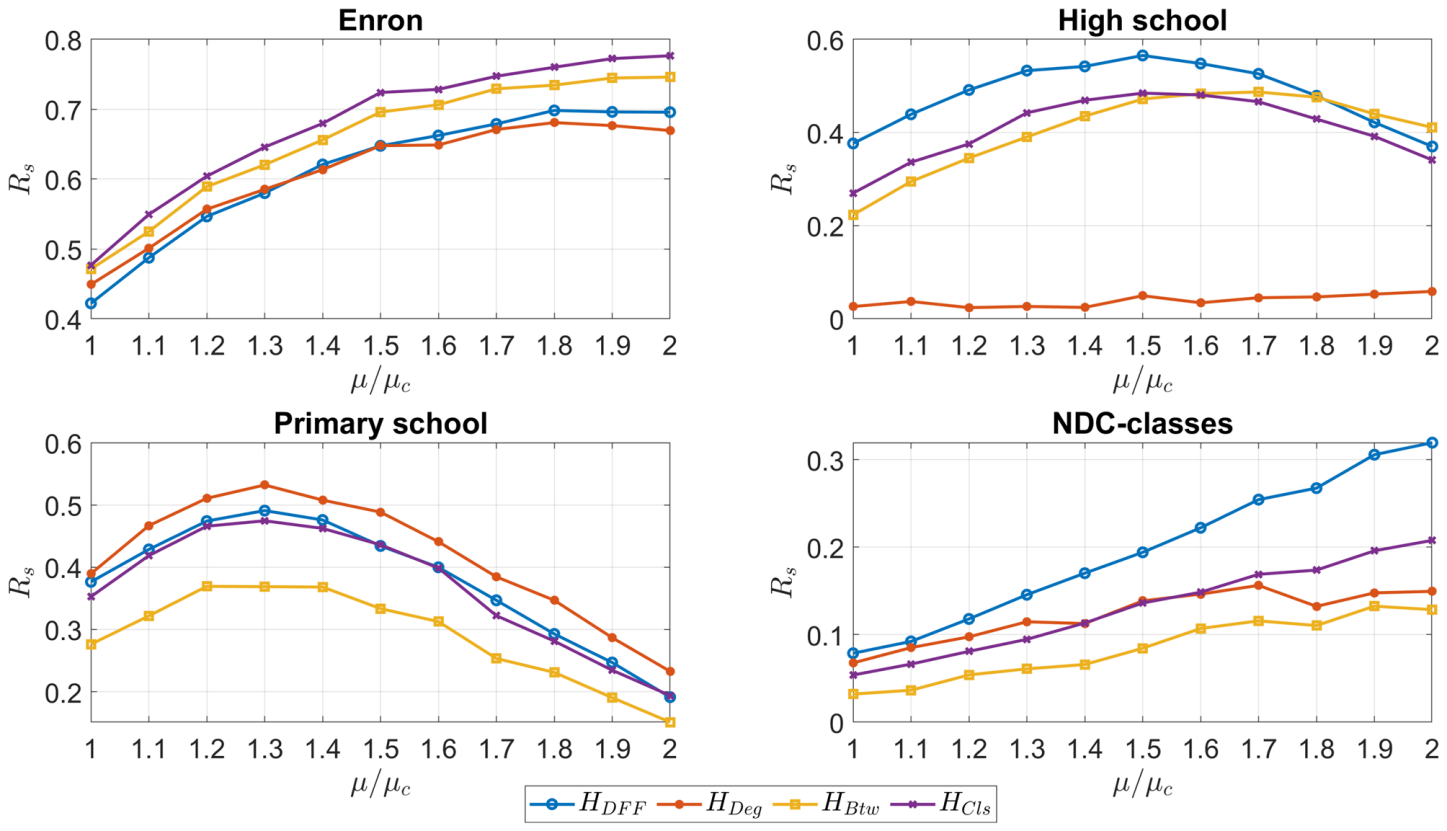
Varying infection rate when using the SIR model on the selected networks. The ratio of hyperedges removed in each trial is the top 5% of influential hyperedges found by each of the centrality measures. A greater diffusion index or $R_s$ indicates that the method is more effective.

As the second experiment, we fix the ratio of the removed higher-order interactions at $5\%$ and vary the infection rate $\mu$, by varying the factor multiplied by $\mu _c$, to compare the centrality measures further in Fig. 3. A greater diffusion index or $R_s$ indicates that the removed hyperedges are influential, and therefore, the method is more effective. With the Enron dataset, closeness centrality performs the most effectively with betweenness centrality after it. DFF overall performs about as well as degree centrality but is outperformed by betweenness centrality and closeness centrality. However, the differences in performances are not drastic, and all the centrality measures overall perform well. In the Primary school network, degree centrality outperforms all the other centrality measures. Closeness and DFF overlap at several infection rates, with DFF performing slightly more effectively at other infection rates. In the High school dataset, DFF outperforms all the other centrality measures. However, after the infection rate reaches 1.8, betweenness centrality performs better than the rest of the centrality measures. Closeness centrality also performs well. In this dataset, degree centrality does not perform as well. When analyzing the NDC-classes network, it can be concluded that DFF outperforms all the centrality measures. Degree centrality performs better than closeness centrality at the lower infection rates. The two centrality measures’ effectiveness is about the same when the infection rate is set in between 1.4 and 1.6. At the greater infection rates, closeness outperforms degree centrality. Betweenness centrality is the least effective of the centrality measures in this dataset, but performance is not drastic. It can be noted that with Enron and NDC-classes, as the infection rate increases, the diffusion index or $R_s$ increases as well. However, for High school and Primary school, $R_s$ initially increases but then decreases. This is due to the network structure of these two datasets. These networks have a large number of hyperedges per vertex comparing the other networks. As a result, the change in the weights is larger than the other networks when 5% of hyperedges are removed and the critical value of the function of the difference between the infection rates before removing and after removing is smaller. For High school and Primary school networks, when we increase $\mu /\mu _c$ from 1 to 2, the difference function reaches the critical value and starts decreasing after this point. For the other two networks, this is not the case. Overall, the centrality methods, with varying performances depending on the network applied to, effectively identify influential higher-order interactions in complex networks for various infection rates used in the SIR model.

## Discussion

By proposing two new hypergraph Laplacians, we are able to generalize DFF, betweenness centrality, closeness centrality, and degree centrality hypergraphs to determine the influential higher-order interactions of a network. These centrality measures are applied to four real-world network datasets. The performances of the centrality measures in identifying influential higher-order network interactions are compared and evaluated by the size of giant component and the SIR model and using spearman’s rank correlation coefficients. Overall, all the centrality measures, adjusted to work with higher-order hyperedges, are effective in finding influential higher-order interactions. The high spearman correlations values for the centrality measures indicate this effectiveness as in Table 3. We also study the role of first-order interactions (edges) in the diffusion process compared to higher-order interactions using both evaluation methods. Our experimental results show that higher-order interactions play more critical roles than first-order interactions. Furthermore, the proposed methods are effective when varying the ratio of the removed influential higher-order interactions and when varying infection rates.

As mentioned earlier, there are not many known centrality measures capable of effectively analyzing and identifying influential higher-order interactions in complex networks. Being able to utilize several centrality measures provides more flexibility, expanding the uses of determining significant higher-order interactions. It also provides more options to select the best possible method of analyzing higher-order interactions based on specific types of networks as well as computational complexity and more room to expand on methods for finding influential higher-order interactions. The results found are significant as they provide a basis for DFF, betweenness centrality, closeness centrality, and degree centrality, through hypergraph Laplacians, to be utilized in real-life applications such as rumor controlling, marketing, disease spreading, advertising, and more.

## Methods

In this section, we start with defining the graph Laplacian. We then present our two hypergraph Laplacians that allow detecting the influential higher-order interactions. Lastly, we present the redefined graph centrality measures. We conclude this section with an illustrative example.

Let *G* be a weighted undirected graph. We define the graph *Laplacian* *L* as $L=D-A$, where *D* is the weighted degree matrix and *A* is the weighted adjacency matrix. The graph Laplacian only uses pairwise interactions, i.e., edges, between vertices and ignores higher-order interactions. Furthermore, it only allows to model diffusion between vertices, not higher-order structures.

To address these concerns, we first represent a complex network with a *hypergraph*. A *hypergraph* *H* denoted by $H=(V,E=(e_i)_{i \in I})$ on the finite vertex set *V* is a family $(e_i)_{i \in I}$ (*I* is a finite set of indexes) of subsets of *V* called *hyperedges*. In a hypergraph, nodes represent entities and hyperedges represent higher-order interactions in the network. The *size* of a hyperedge is the number of the nodes in the corresponding higher-order interaction.

We use the diffusion framework on hypergraphs for identifying critical higher-order interactions. In this work, we model diffusion over a hypergraph inspiring from the the simplicial Laplacians defined in Horak et al.^37^. In the simplicial Laplacians, a hyperedge of size $k+1$ is called a *k*-simplex. For example, vertices are called 0-simplices, edges are called 1-simplices and triangles are called 2-simplices. Let $D_p \in \mathbb {R}_2^{n_{p+1}} \times \mathbb {R}_2^{n_p}$ be the *incidence matrix* that encodes which *p*-simplices are incident to which $(p+1)$-simplices where $n_p$ is number of *p*-simplices. It is defined as

2 $$\begin{aligned} D_p(i,j)= \left\{ \begin{array}{ll} 1 &\qquad{} if \,\sigma _j^p\, is \,on\, the\, boundary \,of \,\sigma _i^{p+1} \\ 0 &\qquad{} otherwise \end{array}\right. \end{aligned}$$

where $\sigma _j^p$ is the *j*-th *p*-simplex. Let $W_p \in \mathbb {R}^{n_p} \times \mathbb {R}^{n_p}$ be the diagonal weight matrix of the *p*-simplices. Then, the *i*-dimensional up Laplacian, $\mathcal {L}_i^{\text {up}} \in \mathbb {R}^{n_i} \times \mathbb {R}^{n_i}$, can be expressed as the matrix

$$\begin{aligned} \mathcal {L}_i^{\text {up}}=W_i^{-1}D_i^{T}W_{i+1}D_i. \end{aligned}$$

Similarly, the *i*-dimensional down Laplacian, $\mathcal {L}_i^{\text {down}} \in \mathbb {R}^{n_i} \times \mathbb {R}^{n_i}$, can be expressed as the matrix

$$\begin{aligned} \mathcal {L}_i^{\text {down}}=D_{i-1}W_{i-1}^{-1}D_{i-1}^{T}W_i. \end{aligned}$$

Lastly, the *i*-dimensional Laplacian in both directions, $\mathcal {L}_i^{\text {both}} \in \mathbb {R}^{n_i} \times \mathbb {R}^{n_i}$, is

$$\begin{aligned} ~ \mathcal {L}_i^{\text {both}}=\mathcal {L}_i^{\text {up}}+\mathcal {L}_i^{\text {down}}. \end{aligned}$$

We now define the proposed hypergraph Laplacians to find the critical higher-order interaction in hypergraphs. To be consistent with the simplicial Laplacian definition, we prefer to call a hyperedge of size $k+1$ as *k*-simplex while defining our Laplacians. We first update the incidence matrix in (2) for simplices of any dimension as follows.

$$\begin{aligned} D_{p,r}(i,j)= \left\{ \begin{array}{ll} 1 &\quad{} if\, \sigma _j^p\, is\, on\, the\, boundary\, of \,\sigma _i^r \\ 0 &\quad {} otherwise \end{array}\right. \end{aligned}$$

for $p<r$ with $\sigma _j^p$ being the *j*-th *p*-simplex. Here, $D_{p,r}$ encodes which *p*-simplices are incident to which *r*-simplices. Next, for a hypergraph with the maximum simplex dimension of *n* (i.e., hyperedge size of $n+1$), Laplacian between *k*-simplices through other simplices, $\mathcal {L}_{k} \in \mathbb {R}^{n_k} \times \mathbb {R}^{n_k}$, is defined as

$$\begin{aligned} \mathcal {L}_{k}=\mathcal {L}_{k,0}+\mathcal {L}_{k,1}+\cdots +\mathcal {L}_{k,n-1}+ \mathcal {L}_{k,n} \end{aligned}$$

for $$k\in \{0,\dots ,n\}$$, where

$$\begin{aligned} \mathcal {L}_{k,l}=\left\{ \begin{array}{ll} W_l^{-1}D_{k,l}^{T}W_{k}D_{k,l} &{} \qquad if \,\,k\le l \\ D_{l,k}W_{k}^{-1}D_{l,k}^{T}W_l &{}\qquad if \,\,k>l \end{array}\right. \end{aligned}$$

with $W_k$ being the diagonal weight matrix of *k*-simplices. Here, $\mathcal {L}_k$ encodes how *k*-simplices are related to each other where the relations can be through the shared neighboring simplices of any dimension. Next, we improve this Laplacian by considering relations between simplices of any dimension. Using the Laplacian $\mathcal {L}_k$, we define the generalized hypergraph Laplacian, $\mathcal {L}_H \in \mathbb {R}^{|E|} \times \mathbb {R}^{|E|}$ with |*E*| being the number of hyperedges in *H*, as the following block matrixwhere $\mathcal {D}_{p,r}=\sum _{i=0}^n D_{p,r}^i$ with $p<r$ and $D_{p,r}^q(i,j)= s$, and *s* is the number of the *q*-simplices that are adjacent to both $\sigma _j^p$ and $\sigma _i^r$ for $q \notin \{p,r\}$ and $D_{p,r}^p=D_{p,r}^r=D_{p,r}$. Here, the blocks on the main diagonal are the Laplacians we develop initially, i.e., they provide the relation between simplices of fixed dimension through simplices of any dimension. Besides, the off-diagonal blocks do the same thing but for different dimensions. Therefore, $\mathcal {L}_H$ is able to capture the relations *between* all simplices *through* simplices of any dimension, which addresses all the limitations.

To compute the influence of the higher-order interactions (i.e., hyperedges), we redefine four graph centrality measures, namely diffusion Frechet function (DFF)^8,43^, degree, betweenness, and closeness^5^, which are originally defined for vertices, to hyperedges, thanks to the generalized Laplacian $\mathcal {L}_H$. We use this Laplacian to model relations between hyperedges, and define the centrality measures accordingly.

DFF centrality employs the diffusion Fréchet function (DFF) defined as the weighted sum of the diffusion distance between a hyperedge and the rest of the network. The diffusion distance measures the similarity between two given hyperedges by finding the similarity of the heat diffusion on a given time interval when the heat source is located on these hyperedges. More formally, let $\mathcal {E}=[\mathcal {E}_1,\ldots , \mathcal {E}_n]^T \in \mathbb {R}^n$ be a probability distribution on the hyperedge set *E* of a hypergraph *H*. For $t>0$, the diffusion Fréchet function on a hyperedge $e_i \in E$ is defined as

$$\begin{aligned} F_{\mathcal {E},t}(i)=\sum _{j=1}^n d_t^2(i,j)\mathcal {E}_j. \end{aligned}$$

with

$$\begin{aligned} d_t^2(i,j)=\sum _{k=1}^n e^{-2\lambda _kt}(\phi _k(i) - \phi _k(j))^2 \end{aligned}$$

where $0\le \lambda _1 \le \cdots \le \lambda _n$ are the eigenvalues of the hypergraph Laplacian $\mathcal {L}_H$ with orthonormal eigenvectors $\phi _1,\ldots ,\phi _n$. A hyperedge with a smaller diffusion Fréchet function value is considered an influential hyperedge in the network since the heat diffusion centered at this hyperedge is similar to many hyperedges. The degree centrality is the number of connections of each hyperedge. The degree can be computed considering the connection weights. The betweenness centrality measures how often each hyperedge appears on the shortest path between two hyperedges in the hypergraph. Since there can be several shortest paths between two hyperedges *s* and *t*, the centrality of hyperedge *u* is

$$\begin{aligned} \displaystyle H_{Btw}(u)=\sum _{s,t \ne u} \frac{n_{st}(u)}{N_{st}} \end{aligned}$$

where $n_{st}(u)$ is the number of shortest paths from *s* to *t* that pass through hyperedge *u*, and $N_{st}$ is the total number of shortest paths from *s* to *t*. Shortest paths can be computed by considering the connection weights. Lastly, the closeness centrality uses the inverse sum of the distance from a hyperedge to all other hyperedges in the hypergraph. The centrality of a hyperedge *u* is

$$\begin{aligned} \displaystyle H_{Cls}(u)=\frac{1}{C_u} \end{aligned}$$

where $C_u$ is the sum of the distances from hyperedge *u* to all other hyperedges. The distance from a hyperedge to another hyperedge can be computed by considering the connection weights.

Lastly, we model SIR on vertices of the hypergraph by utilizing the Laplacian $\mathcal {L}_{0}$ for evaluation. As we mentioned before, $\mathcal {L}_{0}$ reveals how vertices are connected through hyperedges of any size.

We now provide an illustrative example of the proposed methods.

**Figure 4:**
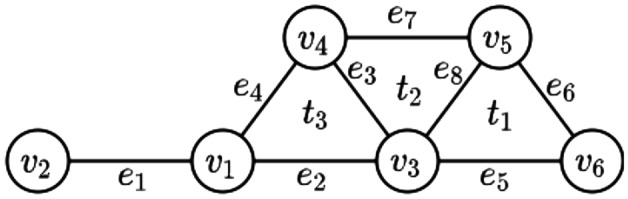
A hypergraph with six vertices (0-simplices), eight edges (1-simplices) and three triangles (2-simplex).

### Example 1

The hypergraph in Fig. 4 has six vertices (0-simplices), eight edges (1-simplices), and three triangles (2-simplices). Its Laplacian between 0-simplices, $\mathcal {L}_{0}$, and generalized Laplacian, $\mathcal {L}_{H}$, are found below. In both Laplacians, the diagonal entries show the number of neighboring simplices for each *k*-simplex (we also count each hyperedge as its neighboring hyperedge in order to stress the importance of the direct neighborhood relation), and the off-diagonal entries show the number of the shared neighboring simplices with other simplices. The diffusion between simplices happens based on the number of the shared neighboring simplices with other simplices in these Laplacians.

**Table 4:** The cells for the best result in each row is colored gray.

	$\mathbf {h}_{dff}$		$\mathbf {h}_{deg}$		$\mathbf {h}_{btw}$		$\mathbf {h}_{cls}$		$\mathbf {r}_s$	
	Rank	Score	Rank	Score	Rank	Score	Rank	Score	Rank	Score
$e_1$	11	1.412	11	7	6	0.026	11	0.533	4	0.078
$e_2$	6	1.218	6	20	2	0.054	3	0.762	5	0.057
$e_3$	4	1.123	4	28	3	0.034	2	0.800	11	0.029
$e_4$	9	1.245	9	18	5	0.026	7	0.696	6	0.052
$e_5$	8	1.231	8	19	9	0.017	8	0.696	7	0.045
$e_6$	10	1.259	10	17	11	0.004	10	0.615	8	0.044
$e_7$	7	1.231	7	19	10	0.015	9	0.696	9	0.040
$e_8$	5	1.123	5	28	4	0.028	4	0.762	10	0.031
$t_1$	3	1.092	3	31	8	0.021	6	0.727	2	0.125
$t_2$	1	1.061	1	34	7	0.024	5	0.762	3	0.111
$t_3$	2	1.081	2	32	1	0.065	1	0.800	1	0.139

To find the influential higher-order interactions, we apply the SIR model and calculate centralities using $\mathcal {L}_0$ and $\mathcal {L}_H$, respectively. As we see in Table 4, $t_1, t_2, t_3$ are the most influential higher-order interactions based on $H_{DFF}$ and $H_{Deg}$ and these results are aligned with the corresponding diffusion index $R_s$. Similarly, $t_3$ is the most influential based on $H_{Btw}$ and $H_{Cls}$. On the other hand, although $e_3$ can be considered as one of the most central and, as a result, influential higher-order interaction, its diffusion index is relatively low. The reason for this is that its neighboring simplices, $v_3, v_4, t_2, t_3$, are of high degree; hence, its removal relatively affects the diffusion.
